# P01-041 – Patient management and rare FMF symptoms

**DOI:** 10.1186/1546-0096-11-S1-A44

**Published:** 2013-11-08

**Authors:** MM Moradian, H Hayrapetyan, G Amaryan, A Yeghiazaryan, T Sarkisian

**Affiliations:** 1 Department of Molecular Genetics and Diagnostics, Morava Inc, Los-Angeles, United States, Yerevan, Armenia; 2 Center of medical genetics and primary health care, Yerevan, Armenia; 3 Arabkir Medical Center, Yerevan, Armenia

## Introduction

Analysis of various symptoms from 20000 FMF patients indicates that several issues, including the clinical manifestation in a variety of combinations and the genotype penetrance, make FMF diagnosis and management challenging. Severe phenotypes with development of serositis, ELE, splenomegaly, and vasculitis are associated with high penetrance mutations of exon 10, mainly M694V allele.

## Objectives

Several forms of arthritis were associated with FMF and the life-threatening complications such as adhesive intestinal obstruction were present in some patients as the first and only manifestation of FMF. Family studies revealed the personalized nature of FMF symptoms and treatment based on genotype and other environmental factors. A significant number of patients have to endure pain for a long time before being properly diagnosed or treated. A major cause for such complexity could be the presence of less common FMF symptoms such as fibromyalgia or multiple sclerosis.

## Methods

Phenotype-genotype correlation was performed after molecular-genetic testing of FMF patients and their family members.

## Results

FMF patient management may seem straightforward due to general response to colchicine therapy yet many issues including but not limited to misdiagnosis, delayed diagnosis, and renal amyloidosis could potentially complicate the patient management. We have observed a limited response to colchicine at the nephrotic stage of renal amyloidosis in FMF patients homozygous for M694V mutation. Almost all such patients have SAA1 a/a genotype suggesting that homozygous M694V patients could benefit from SAA1 genotyping for colchicine dosage adjustment and management of renal amyloidosis. FMF patients with other genotypes had a good chance to ameliorate the nephrotic syndrome and to maintain renal function. Presence of only one symptom in FMF has been a major factor for misdiagnosis and delay in treatment further emphasizing that mutations in MEFV gene could result in various forms and combinations of symptoms in different individuals. Diagnosis of member of family with FMF while his father and sister had the same genotype and no symptoms also points to a personalized development and progression of the disease. Therefore each case should be discussed in details using genotype and symptoms correlation and treated accordingly.

## Conclusion

In our experience prevention of the attacks has been a useful tool in patient management. Abrupt and extreme changes in weather patterns, strenuous activity or exercise, anxiety and stress, and even diet have triggered FMF attacks. These conditions either cause additional inflammation or lower/distort the effectiveness of Pyrin in patients with affected genotypes. Although colchicine therapy remains the dominant treatment for FMF and largely prevents the development of renal amyloidosis, its effect in some cases remains controversial. Of course for genotypes with potential for amyloidosis analysis of SAA1 gene could provide preventive values. Once the diagnosis is final immediate initiation of colchicine therapy could prevent renal complications yet emphasis on identifying environmental and/or social factors that trigger FMF attacks could reduce their frequency and facilitate patient management.

## Disclosure of interest

None declared.

